# Surgical tumour excision of pleomorphic adenoma of submandibular salivary gland: A case report and literature review

**DOI:** 10.1016/j.ijscr.2023.108236

**Published:** 2023-04-25

**Authors:** Msafiri Birigi, Clement N. Mweya

**Affiliations:** a Dental Department, Mbeya Zonal Referral Hospital, P.O. Box 419, Mbeya, Tanzania; b Mbeya College of Health and Allied Sciences, University of Dar es Salaam, P.O. Box 608, Mbeya, Tanzania; c Mbeya Medical Research Centre, National Institute for Medical Research, P.O. Box 2410, Mbeya, Tanzania

**Keywords:** Surgical tumour excision, Pleomorphic adenoma, Submandibular salivary gland, Quality of life, Case report, Tanzania

## Abstract

**Introduction and importance:**

Pleomorphic adenoma is a benign tumour commonly found in salivary glands and surgical excision is the preferred treatment.

**Case presentation:**

This case report describes a 37-year-old male patient who was psychologically affected, socially stigmatised and economically jeopardised, underwent successful surgical excision of a pleomorphic adenoma in the submandibular gland. The procedure involved making a lower submandibular incision through the skin, followed by blunt dissection to approach the tumour, where the whole gland was involved and excised. Haemostasis was achieved through compression, transfixing, and under-running sutures. The patient recovered well without any complications and was discharged with a good outlook.

**Clinical discussion:**

Proper diagnosis and using proper surgical techniques is essential to ensure favourable outcomes. Early diagnosis and prompt management of pleomorphic adenoma are crucial to prevent complications and improve the patient's quality of life.

**Conclusion:**

Surgical tumour excision is a safe and effective treatment for pleomorphic adenoma in the submandibular gland. Successful surgery has significant changes in the quality of life of the patient.

## Highlights

• Pleomorphic adenoma is a common benign tumour of salivary glands and surgical excision is the preferred treatment.
• A case report describes the successful surgical excision of pleomorphic adenoma in a 37-year-old male patient's.
• The procedure involved making a lower submandibular incision through the skin, followed by blunt dissection to approach the tumour, where the whole gland was involved and excised.
• The patient recovered without complications and was discharged with a good outlook.
• Successful surgery has significant changes in the quality of life of the patient.

## 1 Introduction

Pleomorphic adenoma, also known as a benign mixed tumour, is the most common benign tumour of the salivary gland, accounting for approximately 60–70 % of all salivary gland tumours [1], [2], [3], [4]. These tumours are more commonly seen in women and have a peak incidence in the fifth and sixth decades of life [5]. The submandibular gland is the second most commonly affected after the parotid gland [6], [7]. Surgical tumour excision is the treatment of choice for pleomorphic adenomas of the salivary gland, with high success rates and low recurrence rates [15]. The procedure removes the tumour while leaving the surrounding glandular tissue intact [5], [8], [9].

Surgical tumour excision is generally well-tolerated with low rates of morbidity. However, certain risks and potential complications may occur, such as bleeding, infection, nerve injury, and recurrence [10], [11]. The risks and benefits of the procedure should be discussed with the patient prior to surgery. However, early diagnosis and prompt surgical management of pleomorphic adenomas of the submandibular gland can result in successful outcomes with minimal morbidity. Accurate diagnosis, proper diagnosis, and a skilled surgical team are all important factors in achieving optimal outcomes. Significant changes in the quality of life have been reported following orofacial surgeries [12], [13]. This case was reported in line with the SCARE 2020 Guideline [14].

## 2 Presentation of case

**Fig. 1:**
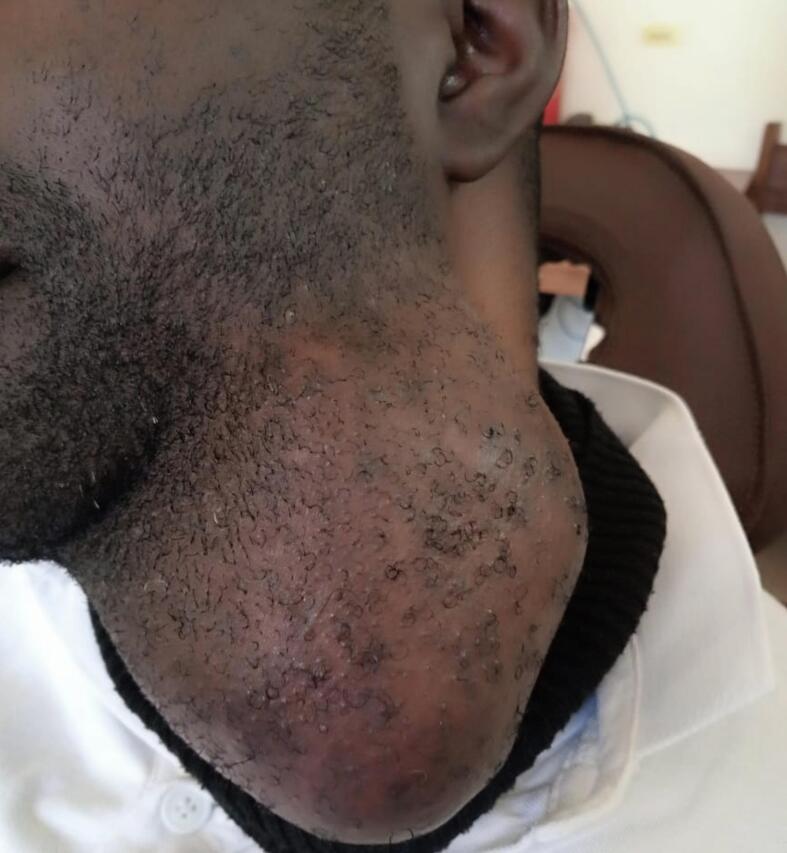
Patient appearance and tumour before the operation.

A 37-year-old male patient reported at the hospital with a chief complaint of swelling on the left side of the lower jaw and the neck for 15 years. The history of present illness (HPI) indicated that the patient was well until 15 years ago when he noticed a painless swelling on the left side of the lower jaw. The swelling started as a small nodule and progressively increased with time. The swelling extended from the lower jaw to cover the neck below to the clavicle on the same side. The patient could not turn his neck for eight years. The swelling was not associated with any aggravating or relieving factors. All that period of illness, the patient visited several health facilities and was given different medications. Traditional healers also treated him without relief. He was wrapping himself around the neck and chest to hide the swelling due to the social stigma. His socio-economic stability was also impaired, as he could not interact with people. He was finally advised to come to the hospital to seek to manage his problem on 4th August 2022. The patient's past medical history (PMH) indicated no previous major surgery or any chronic disease like diabetes mellitus, hypertension, HIV/AIDS, TB and blood transfusion. The patient has no known allergy to food, drug, or substance. The patient reported a history of visiting a dentist due to the same condition. Family and social history indicated that the patient is married, a businessman living with his family, does not drink alcohol, neither smoke cigarettes nor chew any tobacco, and has no family member with the same disease. Upon review of other systems, the patient had no abnormality detected (Fig. 1).

A general examination of the patient indicated being fully conscious with a GCS of 15/15, well oriented to people, place and time, not jaundiced, not pale, no cyanosis, no palpable lymph nodes, both central and peripheral. The vital signs were normal, blood pressure 118/64 mm Hg, respiratory rate 17 breaths/min, temperature 36.6 °C, pulse rate 90 beats/min, with good volume, regular rhythm, non-collapsing and synchronous with other peripheral pulses. On oral examination, the patient had facial asymmetry due to a swelling on the left side of the lower jaw and neck. The swelling extended from the lower border of the body of the mandible down to the lower side of the neck with a diameter of 12 cm posteroanteriorly and with a diameter of 20 cm superoinferiorly. The overlying skin was normal in colour and texture, neither ulcerated nor fixed to the swelling. The swelling was noduleated, mobile, not fixed to the underlying tissues, firm in consistency, non-tender on both superficial and deep palpation. The temporomandibular joint examination found that the jaw was opened to its maximum level with no pain, no cracking sounds, no mouth deviation on opening and closing the mouth. On intraoral examination, the patient had normal lips and competent, normal oral mucosa, palate, gums and tongue. On examination of the dentition, all teeth were present in the mouth, with no dental caries, mobile teeth, periodontal pockets or filled teeth. The provisional diagnosis was pleomorphic adenoma of the left submandibular gland. Routine investigations were performed and found normal, such as full blood picture and blood grouping and cross-matching. The incisional tissue biopsy confirmed the diagnosis of pleomorphic adenoma of the salivary gland.

**Fig. 2:**
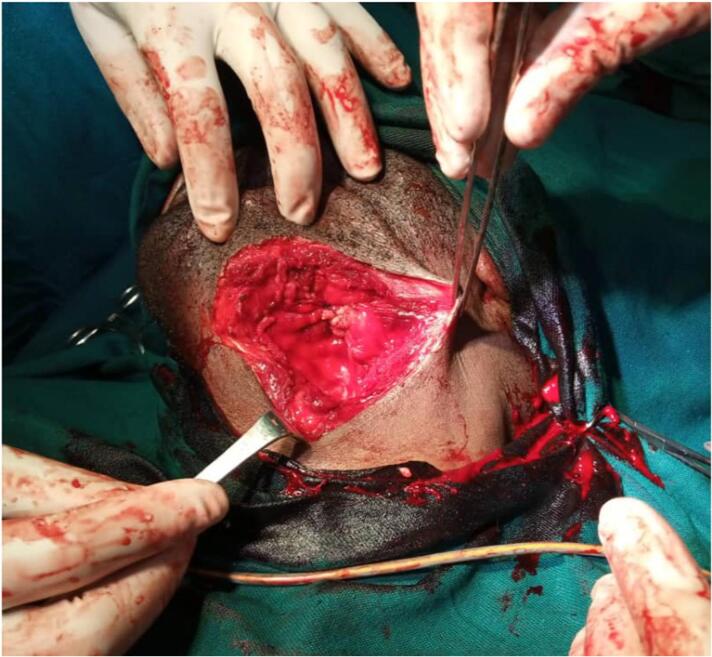
The wound after tumour excision, intra-operative.

**Fig. 3:**
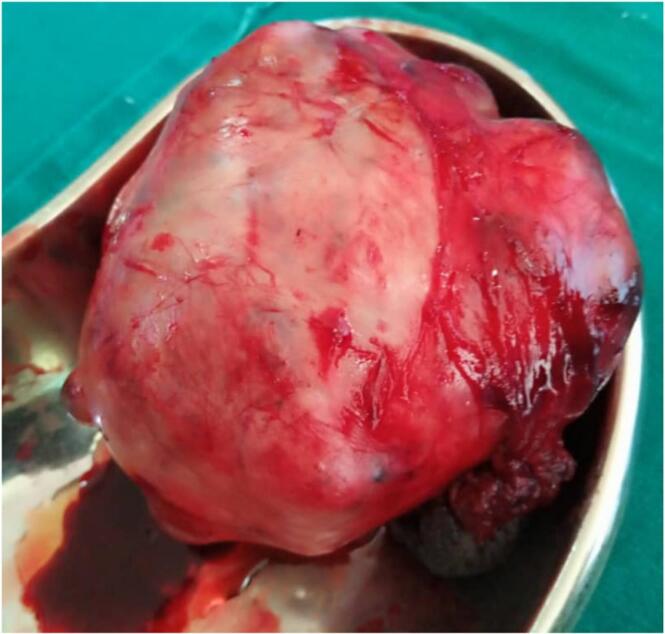
Tumour mass after excision.

The patient was admitted and prepared for surgical tumour excision under general anaesthesia. The preoperative anaesthetist review found that the patient was fit/eligible for general anaesthesia. The preoperative medications were given before surgery. Intraoperatively, the patient was placed in a supine position and orally intubated. The surgical site was cleaned as per standard operating procedures. The jungle juice was injected around the tumour mass to minimize bleeding. The twelve centimetres lower submandibular incision was made through the skin at the middle of the swelling, posteroanterior, from the level of the posterior aspect of the sternocleidomastoid muscle to the level of the symphysis menti. The blunt dissection was used to approach the tumour, and the whole submandibular gland was removed and taken for tissue re-biopsy, of which the results were the same as the preoperative incisional biopsy. Compression, transfixing and under-running sutures achieved haemostasis. The wound was closed by layer using vicryl 2.0, and the skin was apposed using nylon 3.0 (Fig. 2, Fig. 3).

**Fig. 4:**
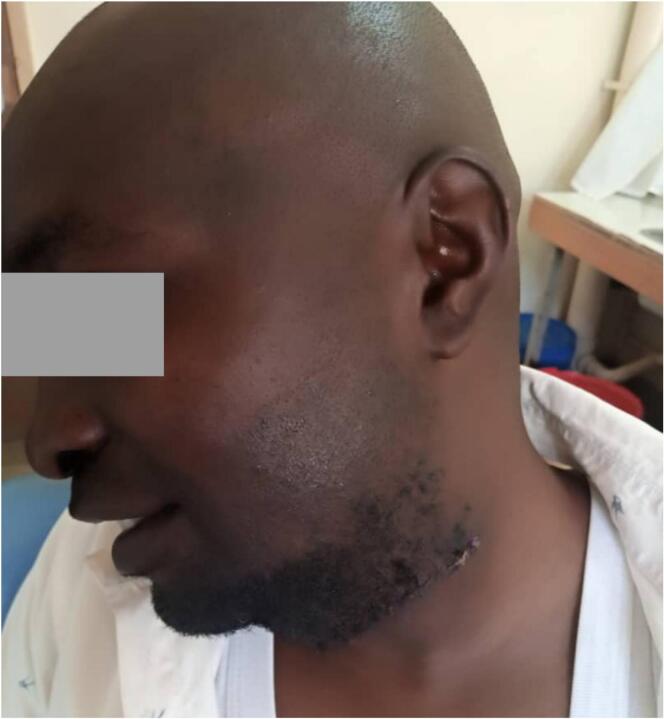
The appearance of the patient ten days post-operative.

**Fig. 5:**
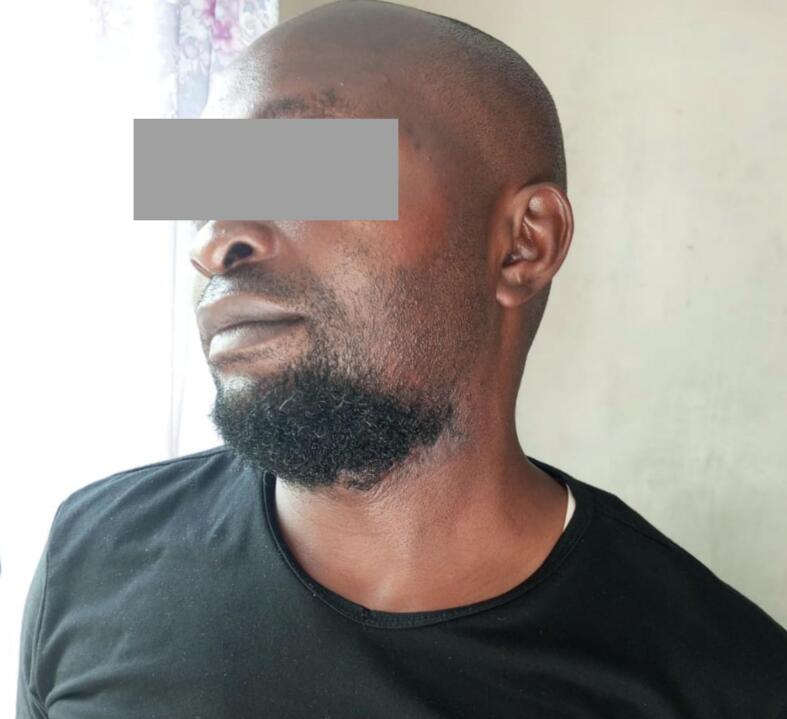
The appearance of the patient three months post-operative.

The patient was awake and stable and post-operative orders were initiated immediately. A post-operative review showed that the patient was awake, stable, able to walk after 24 h without any complications. The wound was clean and had no oedema. Therefore, the patient was discharged. The sutures were removed after ten days of post-operative, he had no new complaints and the wound was clean and dry. The healing process was progressing well. After 21 days of post-operative, the superficial wound healing was complete, and there were no complaints from the patient. The patient had no complications three months post-operative and continued his daily activities happily. The patient reported being happy and has resumed his daily business, free from social stigma. He says his quality of life was jeopardised, and he has now rejuvenated and feels like a born-again person. The patient will be follow-up after every six months for five years (Fig. 4, Fig. 5).

## 3 Discussion

Post-operative reviews play an essential role in evaluating the success of surgical procedures. The patient's post-operative review showed no complications or complaints, indicating a successful surgical procedure [16]. The patient's ability to resume daily activities after just 24 h is a positive outcome, indicating a faster recovery time, often associated with better post-operative outcomes [17]. The success of the surgical excision procedure can be attributed to several factors. First, the surgical technique was likely minimally invasive, allowing faster healing. Second, the patient's overall health and well-being before the surgery could have significantly affected the speedy recovery [17]. Additionally, post-operative care and follow-up patient monitoring are crucial for ensuring continued recovery and identifying potential complications. This includes pain management, infection monitoring and adequate wound care [16].

The absence of oedema and a clean wound after surgery indicates appropriate post-operative wound management and successful surgery. The final post-operative review after three months demonstrating the patient's full recovery and ability to resume daily activities highlights the effectiveness of surgical tumour excision for pleomorphic adenoma of the submandibular salivary gland. Surgical excision was a safe and effective treatment option for benign salivary gland tumours, with a low risk of complications and high success rates [18]. There have been reported significant changes in quality of life following orofacial surgeries. Quality of life has been recognized as an important endpoint in addition to disease-related and global survival. It is particularly important for patients with salivary gland neoplastic disease. For patients undergoing benign salivary gland tumour surgery, cosmetic and functional outcomes are extremely important, as these patients' psychological well-being and ability to function in society can be severely impacted [12], [13]. This was found realistic to our patient, who was marginalised by his community. He could not interact and perform any socio-economic responsibilities. However, after successful tumour excision, he resumed his socio-economic activities with maximum interaction. He is leaving a happy life.

## 4 Conclusion

Surgical tumour excision of pleomorphic adenoma of the submandibular salivary gland is a safe and effective treatment option with low morbidity rates. This case report demonstrates that the procedure can be performed with minimal complications and excellent outcomes. Early diagnosis and prompt surgical intervention are essential to successfully managing this benign tumour. Proper diagnosis and a skilled surgical team are critical to achieving optimal outcomes. With careful preoperative planning, adherence to surgical protocols, and close post-operative monitoring, surgical excision can provide an excellent option for patients with pleomorphic adenoma of the submandibular salivary gland. In this case, the successful outcome of surgery significantly changed the patient's quality of life. The surgery had a vivid psychological and socio-economic impact on the patient. However, continued research is necessary to identify new diagnostic and treatment strategies for these tumours, including minimally invasive and non-surgical options. With ongoing efforts, we can further improve patient outcomes and quality of life for those affected by this common benign tumour.

## Patient (parent's) consent

Written informed consent was obtained from the patient to publish this case report and accompanying images. A copy of the written consent is available for review by the Editor-in-Chief of this journal on request.

## Ethical approval

Ethical approval was provided by the author's institution.

## Funding

None.

## Guarantor

Dr. Msafiri Birigi.

## CRediT authorship contribution statement

Dr. Msafiri Birigi conducted the procedures. Dr. Msafiri Birigi and Dr. Clement N. Mweya were involved in the writing of this case report.

## Conflicts of interest

None declared.
